# TNFSF15 facilitates human umbilical cord blood haematopoietic stem cell expansion by activating Notch signal pathway

**DOI:** 10.1111/jcmm.15626

**Published:** 2020-09-10

**Authors:** Yahui Ding, Shan Gao, Jian Shen, Tairan Bai, Ming Yang, Shiqi Xu, Yingdai Gao, Zhisong Zhang, Luyuan Li

**Affiliations:** ^1^ State Key Laboratory of Medicinal Chemical Biology College of Pharmacy and Tianjin Key Laboratory of Molecular Drug Research Nankai University Tianjin China; ^2^ Key Laboratory of Experimental Hematology Institute of Hematology and Blood Diseases Hospital Chinese Academy of Medical Sciences and Peking Union Medical College Tianjin China

**Keywords:** HSC expansion, Notch signalling pathway, TNFSF15

## Abstract

The lack of efficient ex vivo expansion methods restricts clinical use of haematopoietic stem cells (HSC) for the treatment of haematological malignancies and degenerative diseases. Umbilical cord blood (UCB) serves as an alternative haematopoietic stem cell source. However, currently what limits the use of UCB‐derived HSC is the very low numbers of haematopoietic stem and progenitor cells available for transplantation in a single umbilical cord blood unit. Here, we report that TNFSF15, a member of the tumour necrosis factor superfamily, promotes the expansion of human umbilical cord blood (UCB)‐derived HSC. TNFSF15‐treated UCB‐HSC is capable of bone marrow engraftment as demonstrated with NOD/SCID or NOD/Shi‐SCID/IL2Rgnull (NOG) mice in both primary and secondary transplantation. The frequency of repopulating cells occurring in the injected tibiae is markedly higher than that in vehicle‐treated group. Additionally, signal proteins of the Notch pathway are highly up‐regulated in TNFSF15‐treated UCB‐HSC. These findings indicate that TNFSF15 is useful for in vitro expansion of UCB‐HSC for clinical applications. Furthermore, TNFSF15 may be a hopeful selection for further UCB‐HSC application or study.

## 1 INTRODUCTION

Haematopoietic stem cells (HSC) are capable of self‐renewal and multi‐lineage differentiation, and thus able to propagate and generate mature blood cell types.^1^, ^2^, ^3^ The regenerative potential of HSC is attractive for using these cells for the treatment of certain haematological malignancies and degenerative diseases.^4^, ^5^ Unfortunately, only about 30% of patients could find a human leucocyte antigen (HLA)‐matched adult sibling donor, despite >20 million adult volunteer donors in the National Marrow Donor Program and affiliated registries.^6^ Umbilical cord blood (UCB) serves as an alternative haematopoietic stem cell source and the use of umbilical cord blood (UCB)‐derived HSC is a highly demanded alternative approach because of reduced need for HLA matching, decreased risk of chronic graft‐vs‐host disease (GVHD) despite HLA disparity, relatively convenient acquisition of the cells and retained graft‐vs‐tumour effects.^7^, ^8^ However, currently what limits the use of UCB‐derived HSC is the very low numbers of haematopoietic stem and progenitor cells available for transplantation in a single umbilical cord blood unit, hindering otherwise a great application of these cells in allogeneic transplantation, particularly in adult recipients.^9^, ^10^ Moreover, the low haematopoietic stem and progenitor cell dose given with UCB transplantation patients could have negative effects on outcomes.^11^ The fundamental of overcoming the obstacle in UCB transplantation is increasing the number of HSC in a single UCB unit by ex vivo UCB expansion. Many studies have translated into significant clinical benefits on UCB‐HSC expansion to enhance UCB‐HSC dose over the last decades. However, currently available techniques are likely expanding more progenitors rather than true stem cells.^12^ It is necessary to develop new strategies to expand UCB‐derived HSC ex vivo.

TNFSF15 is a cytokine involved in maintaining vascular homeostasis. This cytokine uniquely exhibits a number of activities: it inhibits neovascularization and vascular hyperpermeability but promotes lymphangiogenesis.^13^, ^14^, ^15^, ^16^, ^17^, ^18^ TNFSF15 can also enhance the survival of bone marrow‐derived Lin^−^Sca1^+^ endothelial progenitor cells in vitro and at the same time inhibit the differentiation of these cells into endothelial cells.^15^ Lin^−^Sca1^+^ mononuclear cells were considered as EPCs, while HSC has the same biomarker. As a result, we speculated that TNFSF15 (VEGI) directly regulated and may inhibit the differentiation HSC. Then, we accidentally discovered TNFSF15 (VEGI) injection by intraperitoneal at 5 mg/kg significantly increased the percentage of Lin^−^‐c‐Kit^+^‐Sca‐1^+^ cells in the bone marrow which suggests that VEGI promotes HSC production. In this study, we investigated the effect of TNFSF15 on UCB‐HSC expansion ex vivo. We found that TNFSF15 was able to stimulate the expansion of UCB‐HSC ex vivo and promote the engraftment of HSC in immune‐deficient mice by activating Notch signal pathway. All the results showed that TNFSF15 was a hopeful selection for further UCB‐HSC application or study.

## 2 MATERIALS AND METHODS

### 2.1 In vitro expansion of human haematopoietic stem cells

Human umbilical cord blood (UCB) samples were obtained from consenting donors at Tianjin Central Hospital of Gynecology Obstetrics (Tianjin, China) according to ethically approved procedures. The informed consent of cord blood collection from pregnant women was signed before the collection of UCB. The UCB collection made according to the Declaration of Helsinki in 1975 by professional gynaecologist. CD34^+^ cells were isolated by immune‐magnetic methods using the MACS CD34 progenitor cell isolation kit (Miltenyi Biotec). The CD34^+^ cell expansion culture medium consisted with Iscove's modified Dulbecco's medium (Life Technologies) supplemented with 10% foetal bovine serum (FBS, Gibco), 100 ng/mL recombinant human stem cell factor (SCF; PeproTech), 100 ng/mL recombinant human thrombopoietin (TPO; PeproTech) and 100 ng/mL recombinant human FMS‐like trysine kinase 3 ligand (Flt3L; PeproTech). The CD34^+^ cells were suspended with expansion medium and seeded into 96‐well plate with 1 × 10^4^ cells/200 µL each well. Following that the recombinant human TNFSF15 or buffer was added at different concentrations. Cells were incubated at 37°C with 5% CO_2_. After treated for 3 or 7 days, the cells were collected and washed with PBS, respectively. Then the cells were resuspended with 100 µL PBS and stained with APC‐conjugated anti‐CD34 (BD; 555824), PE.Cy7‐conjugated anti‐CD38 (BD; 560677), APC.Cy7‐conjugated anti‐CD45RA (BD; 560674), PerCP.Cy5.5‐conjugated anti‐CD90 (BD; 561557) and PE‐conjugated anti‐CD49f (BD; 555736) antibodies for 30 minutes at room temperature in dark. After washed with PBS, the absolute number and phenotype analysis were detected by flow cytometry. Upon treatment with recombinant human TNFSF15 or vehicle, the cells were subjected to characterization with flow cytometry, clonogenic, cobblestone area‐forming and single‐cell assays. The small compounds SR1, UM171 and Notch signal pathway inhibitor DAPT (GSI‐IX) were purchased in Selleck. Each experiment was repeated for three times.

### 2.2 Colony formation assay

Fresh CD34^+^ cells were suspended with in expansion medium and seeded into 96 well plate with 1 × 10^4^ cells/200 µL each well. After treated with TNFSF15 (2 µg/mL, 7 days), 10 µL cell suspension (equivalent to 200 initial cells) was mixed with Methocult H4434 gently and evenly. Then the mixture was plated into 6‐well plate and cultured at 37°C with 5% CO_2_ for 10 days. The number of CFU‐GM, CFU‐E, BFU‐E, CFU‐G, CFU‐M and CFU‐GEMM was scored according to morphological features. The experiment was repeated for three times.

### 2.3 Cobblestone area‐forming cells (CAFC) assay

M_2_‐10B_4_ feeder cells irradiated with 80 Gy using a 137‐Caesium source were plated in collagen‐coated 96‐well flat‐bottom plates (1 × 10^4^ cells per well) in human long‐term culture media (HLTM; MyeloCult H5100, Stem cell Technologies) for 24 hours prior to addition of CD34^+^ cells. Freshly isolated CD34^+^ cells from human umbilical cord blood were treated with TNFSF15 (2 µg/mL) or vehicle for 7 days in expansion medium, then rinsed with PBS, resuspended in 0.1 mL of HLTM containing 1 µmol/L hydrocortisone, and added to the M_2_‐10B_4_ cultures at different cell number concentrations with 12 replicates each concentration. The CAFC cultures were maintained for 5 weeks at 37°C with 5% CO_2_ for five weeks with changing half medium weekly. Cobblestone areas in the cultures were then scored. CAFC frequencies were calculated by using Poisson analysis based on the proportion of negative wells and maximum likelihood. Statistical analysis was performed by using the L‐Calc software for limiting dilution assay.

### 2.4 Differentiation assay

Fresh CD34^+^ cells from human umbilical cord blood were cultured in expansion culture medium for 7 days in the presence of TNFSF15 at 37°C with 5% CO_2_. Then cells were collected, respectively, washed and stained with PE.Cy7‐conjugated anti‐CD3 (BD; 557851), APC.Cy7‐conjugated anti‐CD33 (BioLegend; 366613), FITC‐conjugated anti‐CD19, PerCP‐conjugated anti‐CD56 (BD; 560842) and APC‐conjugated anti‐CD235a (BD; 561775) for 30 minutes under room temperature in dark. Then the cells were resuspended and analysed by flow cytometry.

### 2.5 Single‐cell assay

Freshly CD34^+^ cells were isolated from human umbilical cord blood and stained with APC‐conjugated anti‐CD34 (BD; 555824), PE.Cy7‐conjugated anti‐CD38 (BD; 560677), APC.Cy7‐conjugated anti‐CD45RA (BD; 560674), PerCP.Cy5.5‐conjugated anti‐CD90 (BD; 561557) and PE‐conjugated anti‐CD49f (BD; 555736) antibodies for 30 minutes at room temperature in dark. After washed with PBS, human DAPI^−^CD34^+^CD38^−^CD45RA^−^CD90^+^CD49f^+^ cells freshly isolated from cord blood were individually sorted into round‐bottom 96‐well plates by using flow cytometry (BD Influx cell sorter; Becton Dickinson Biosciences). The cells were cultured in Iscove's Modified Dulbecco's Medium (IMDM; Gibco) supplemented with 15% FBS, 0.25% bovine serum albumin (BSA; Sigma), 100 ng/mL SCF, 100 ng/mL TPO, and 100 ng/mL Flt3L, in the presence or absence of TNFSF15 (2 µg/mL) for 14 days with 60 replicates at 37°C with 5% CO_2_. The number of effect wells with expanded cells and cell number in the presence or absence of TNFSF15 for 7 days was scored under microscope. On day 14, wells with more than 200 cells were stained with APC‐conjugated anti‐CD34 and PE‐conjugated anti‐CD49f antibodies for 30 minutes at room temperature in dark. Then the percentage and absolute number of CD34^+^CD49f^+^ in the single‐cell cultures were detected and calculated using a BD LSR Fortessa cell analyzer. Each experiment was repeated for three times with three umbilical cord blood donors and 60 repeat wells for each group in each experiment.

### 2.6 SRC assay

CD34^+^ cells from human umbilical cord blood were cultured in expansion culture medium for 7 days in the presence of TNFSF15 at 37°C with 5% CO_2_. Six‐week‐old female NOD/SCID (Institute of Laboratory Animals, Beijing, China) was irradiated with X‐ray (200 cGy) 12 hours before transplantation. Then, the cells after treatment of TNFSF15 were collected, washed and calculated. The NOD/SCID mice were randomized and injected with cultured CD34^+^ cells with or without TNFSF15 treatment at different cell concentrations (2000, 5000, 10 000 and 20 000 initialling cells, respectively) intravenously. Bone marrow (BM) cells were collected, stained with FITC‐conjugated anti‐human CD45 (BD, 561865) and analysed for human cell engraftment by flow cytometry in 12 weeks.

### 2.7 NOG mice transplantation

CD34^+^ cells from human umbilical cord blood were stained with APC‐conjugated anti‐CD34 (BD; 555824), PE.Cy7‐conjugated anti‐CD38 (BD; 560677), APC.Cy7‐conjugated anti‐CD45RA (BD; 560674), PerCP.Cy5.5‐conjugated anti‐CD90 (BD; 561557) at room temperature for 30 minutes in dark. Then human CD34^+^CD38^−^CD90^+^CD45RA^−^ cells were sorted and treated with TNFSF15 (2 µg/mL) in expansion culture (300 cells/well) for 4 days at 37°C with 5% CO_2_. NOD/Shi‐SCID/IL2Rg‐null (NOG) mice (Beijing Vital River Laboratory Animal Technologies Co. Ltd) were irradiated with X‐ray (200 cGy) 12 hours before transplantation. Then the cells were collected and injected into the right tibia of the animals with 300 initiating cells/25 µL each NOG mouse by intramedullary injection. The percentage of engraftment was analysed 12 weeks post‐transplantation in the peripheral blood and in the injected side and opposite side of bone marrow by flow cytometry using human CD45 staining. Multi‐lineage differentiation was detected by using antibodies of different linages.

As to secondary transplantation, the receptor NOD/Shi‐SCID/IL2Rg‐null (NOG) mice (Beijing Vital River Laboratory Animal Technologies Co. Ltd) were irradiated with X‐ray (200 cGy) 12 hours before transplantation. Then the bone marrow cells from donor mice (primary engrafted NOG mouse) were injected into the new NOG mice with one donor corresponds to one recipient intravenously. After 16 weeks, the percentage of engraftment was detected using human CD45 staining.

### 2.8 Cell apoptosis assay

Cell apoptosis assay was performed according to the manufacture`s advice. CD34^+^ cells from human umbilical cord blood were cultured in expansion culture medium for 7 days in the presence of TNFSF15 at 37°C with 5% CO_2_. The cells were collected and washed with PBS. Then resuspended with 1× loading buffer, and stained with Annexin Ⅴ‐FITC and PI for 15 minutes at room temperature in dark. After that the cells were analysed by flow cytometry.

### 2.9 Cell cycle assay

CD34^+^ cells from human umbilical cord blood were cultured in expansion culture medium for 7 days in the presence of TNFSF15 at 37°C with 5% CO_2_. The cells were collected, washed with PBS and fixed with cold 70% ethanol overnight. After washed with PBS twice, the cells were incubated with propidium iodide buffer in the dark for 30 minutes. Then cell cycle distribution was determined by flow cytometry. As to G0 phase detection, the cells were collected, fixed with cold 70% ethanol for 1 hour on ice and stained with FITC‐conjugated anti‐Ki67 (eBioscience, 11‐5698‐82) antibody at room temperature in dark for 60 minutes. The cells were incubated with propidium iodide buffer in the dark for 30 minutes. The percentage of G0 phase was determined by flow cytometry.

### 2.10 Single‐cell PCR assay

CD34^+^ cells from human umbilical cord blood were cultured in expansion culture medium for 7 days in the presence of TNFSF15 at 37°C with 5% CO_2_. Then the cells were collected, respectively, and stained with APC‐conjugated anti‐CD34 (BD; 555824), PE.Cy7‐conjugated anti‐CD38 (BD; 560677), APC.Cy7‐conjugated anti‐CD45RA (BD; 560674), PerCP.Cy5.5‐conjugated anti‐CD90 (BD; 561557) and PE‐conjugated anti‐CD49f (BD; 555736) antibodies for 30 minutes at room temperature in dark. Then 50 CD34^+^CD38^−^CD45RA^−^CD90^+^CD49f^+^ cells were sorted into a mixture of CellsDirect 2× Reaction Mix, 0.2× TaqMan Assay Mix (Applied Biosystems) and SuperScript III RT/ PlatinumTaq Mix (Invitrogen). Total RNA was extracted with CellsDirect One‐Step qRT‐PCR Kit (Invitrogen) according to the manufacturer's instructions. Reverse transcription and specific target amplification were performed continuously with the following parameters: 50°C for 15 minutes, 95°C for 2 minutes, 95°C for15 s for 18 cycles and 60°C for 4 minutes. Pre‐amplified cDNA was diluted with TE buffer (1:5), and PCR assay was performed. Data were analysed using BioMark Real‐Time PCR Analysis Software (Fluidigm).

### 2.11 Western blot assay

CD34^+^ cells from human umbilical cord blood were cultured in expansion culture medium for 7 days in the presence of TNFSF15 at 37°C with 5% CO_2_. The cells were collected in lysis buffer. The BCA assay was used to determine the protein concentration. Equal amounts of protein were separated by SDS‐PAGE and transferred to PVDF membranes. After blocked by nonfat milk, the membranes were incubated with antibodies specific for c‐myc, hes1, Notch1, NCID and β‐actin overnight. Then washed with PBST for five times, incubated with anti‐HRP secondary antibody and visualized by the Tanon Chemiluminescent Imaging System.

### 2.12 Statistical analysis

Results are presented as means ± SD *P* values <.05 were considered statistically significant. *P < .05; **P < .01; ***P < .001. Student's *t* test and Nonparametric Mann‐Whitney test were performed using GraphPad Prism 5 (GraphPad software).

## 3 RESULTS

### 3.1 TNFSF15 increases the number of primitive human CD34^+^CD49f^+^ haematopoietic stem cells

**FIGURE 1:**
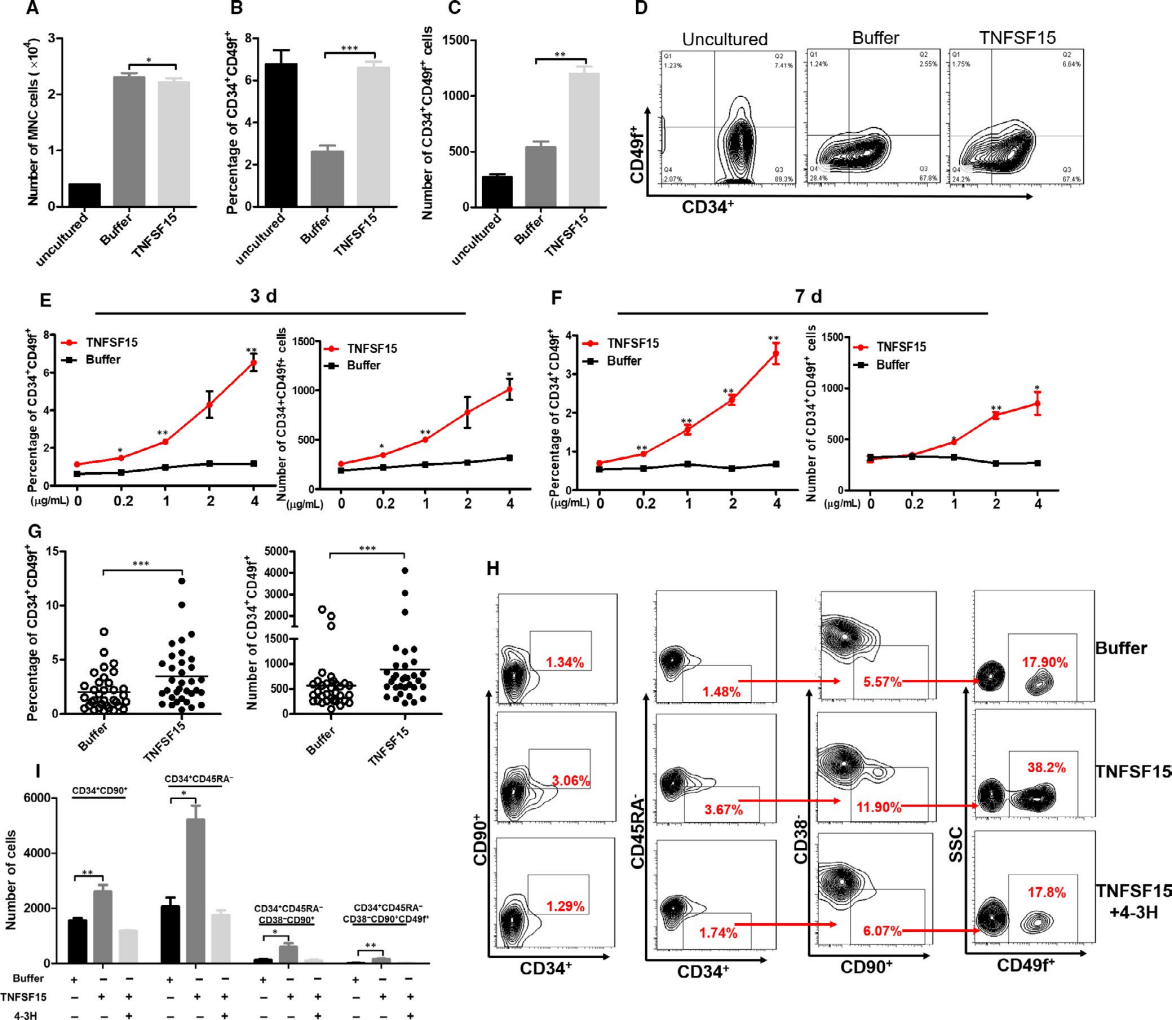
TNFSF15 promotes in vitro expansion of primitive human CD34^+^CD49f^+^ haematopoietic stem cells. A, Number of total mononuclear cells after being treated with TNFSF15 for 7 d at 2 µg/mL in expansion medium (n = 3). B, Percentage of CD34^+^CD49f^+^ cells after being treated with TNFSF15 for 7 d at 2 µg/mL in expansion medium on the same human umbilical cord blood sample (n = 3). 1 × 10^4^ CD34^+^ human UCB cells were seeded in the beginning. The experiment was repeated for three times. C, Absolute number and representative pictures (D) of CD34^+^CD49f^+^ cells after the treatment of TNFSF15 at 2 µg/mL for 7 d in expansion medium on the same human umbilical cord blood sample (n = 3). E, Percentages and absolute number of CD34^+^CD49f^+^ cells in CD34^+^ cells treated with various concentrations of TNFSF15 (0, 0.2, 1, 2 and 4 µg/mL) for 3 d in expansion medium with 1 × 10^4^ initiating CD34^+^ cells. F, Percentages and absolute number of CD34^+^CD49f^+^ cells in CD34^+^ cells treated with various concentrations of TNFSF15 (0, 0.2, 1, 2 and 4 µg/mL) for 7 d in expansion medium with 1 × 10^4^ initiating CD34^+^ cells (n = 3). G, The percentage and total number of CD34^+^CD49f^+^ cells per sample of 33 cases of human umbilical cord blood samples cultured in the presence or absence of TNFSF15 (2 µg/mL) for 7 d in expansion medium (n = 33); horizontal bar, mean value. H, Representative flow cytometry images of CD34^+^CD90^+^, CD34^+^CD45RA^−^, CD34^+^CD45RA^−^CD90^+^CD38^−^ and CD34^+^CD45RA^−^ CD90^+^CD38^−^CD49f^+^ cells after being treated with TNFSF15 (2 µg/mL) in the presence or absence of TNFSF15 and TNFSF15‐neutralizing antibody 4‐3H. Each experiment was repeated for three times. I, The number of CD34^+^CD90^+^, CD34^+^CD45RA^−^, CD34^+^CD45RA^−^CD90^+^CD38^−^ and CD34^+^CD45RA^−^ CD90^+^CD38^−^CD49f^+^ cells after being treated with TNFSF15 (2 µg/mL) in the presence or absence of TNFSF15 and TNFSF15‐neutralizing antibody 4‐3H (n = 3)

Notta and colleagues reported that CD49f was a unique cell surface marker of HSCs that contributed greatly to the separation of HSCs from multi‐potent progenitors (MPPs).^19^ Therefore, we used CD34 and CD49f as HSC enrichment markers to validate the HSC expansion effect. We collected human umbilical cord blood and first isolated CD34^+^ bulk cells for a dose response assay of TNFSF15 and the purity of CD34^+^ cells after magnetic sorting guaranteed at about 95% (Figure S1A). We found that TNFSF15 could significantly increase the percentage and the total number of CD34^+^CD49f^+^ cells with slightly inhibition of total mononuclear cells (Figure 1A‐D). Furthermore, we analysed the expansion effect of TNFSF15 with a dose‐dependent manner for 3 and 7 days, respectively. The results showed that TNFSF15 increased the percentage and the total number of CD34^+^CD49f^+^ cells with a dose‐dependent manner at 3 and 7 days (Figure 1E and F). Furthermore, the HSC expansion capacity of TNFSF15 was confirmed with UCB from 33 individuals (Figure 1G). We then analysed the effect of TNFSF15 on other subpopulations of HSCs by flow cytometry. The result suggested that TNFSF15 also gave rise to a significant increase the percentage and absolute number of CD34^+^CD45RA^−^, CD34^+^CD90^+^, CD34^+^ CD38^−^CD90^+^CD45RA^−^ and CD34^+^CD49f^+^CD90^+^CD45RA^−^CD38^−^ cells (Figure 1H and I). The use of a neutralizing antibody of TNFSF15 (4‐3H) prevented the percentage and absolute number increase of CD34^+^CD45RA^−^, CD34^+^CD90^+^, CD34 CD38^−^CD90^+^CD45RA^−^ and CD34^+^CD49f^+^CD90^+^CD45RA^−^CD38^−^ cells induced by TNFSF15 (Figure 1H and 1I). In the differentiation assay, the presence of SCF, TPO and Flt3L modifies the differentiation capacity with significantly increased frequency of myeloid cell (CD33) and erythroid cell (CD235a) compared with freshly isolated CD34^+^. However, in the culture medium with SCF, TPO, and Flt3L, TNFSF15 treatment did not change the percentage of lymphocyte cell (CD19), T cell (CD3), erythroid cell (CD235a), myeloid cell (CD33) and NK cell (CD56) compared with buffer group which suggested TNFSF15 did not affect the differentiation during the culture (Figure S1B).

### 3.2 TNFSF15 sustained self‐maintenance and multi‐lineage differentiation potential of haematopoietic stem cells

**FIGURE 2:**
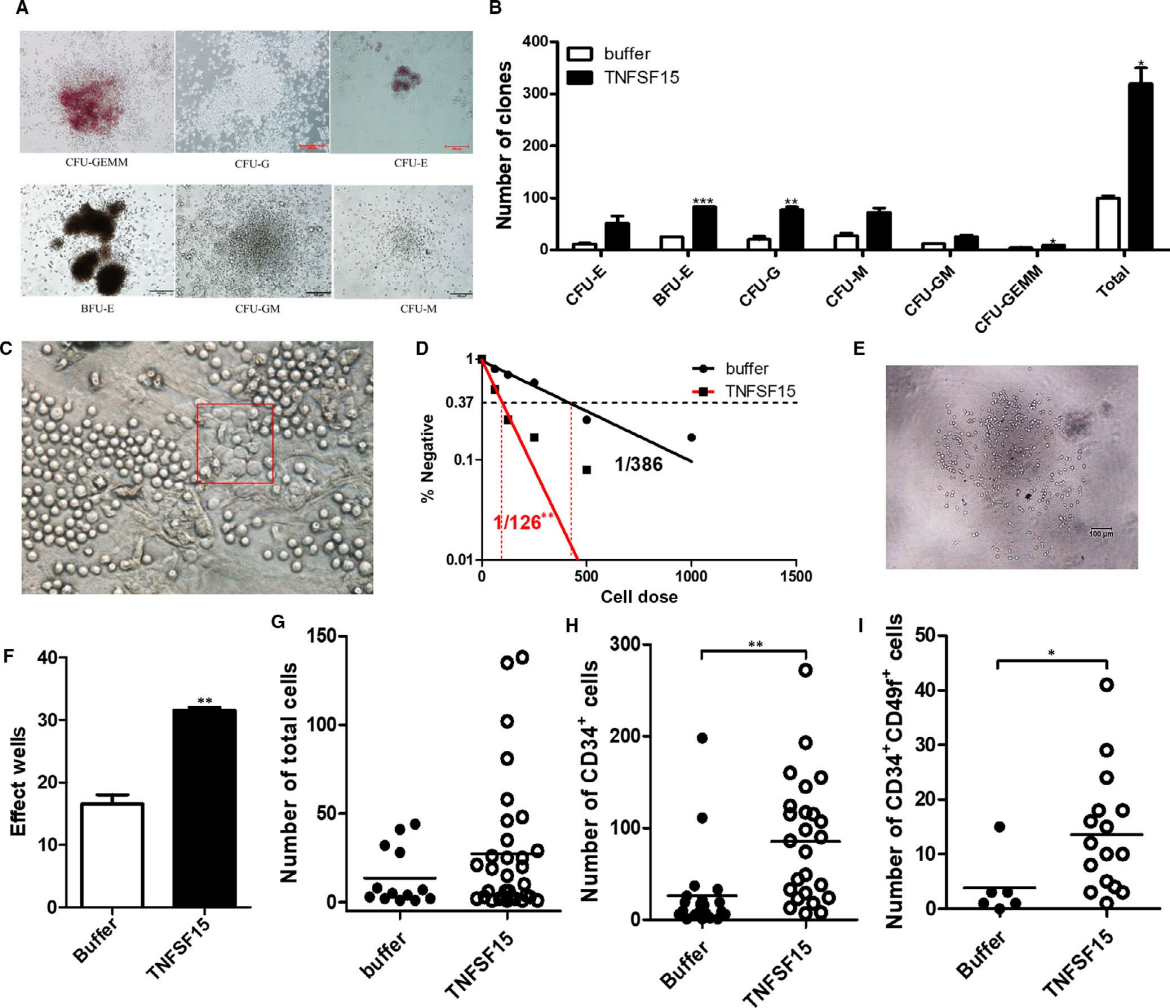
TNFSF15 sustained self‐maintenance and multi‐lineage differentiation potential of haematopoietic stem cells. A, Typical images of representative morphologies of various type of colonies as indicated. B, Colony formation assay showing the number of various types of cell colonies as indicated. The number of colony‐forming unit‐erythroid (CFU‐E), Burst‐forming unit‐erythroid (BFU‐E), Colony‐forming unit‐granulocyte (CFU‐G), Colony‐forming unit‐macrophage (CFU‐M), Colony‐forming unit‐granulocyte, macrophage (CFU‐GM) and Colony‐forming unit‐granulocyte, erythroid, macrophage, megakaryocyte (CFU‐GEMM) were scored after cultured for 10 d H4434 methylcellulose (n = 3). The experiment was repeated for three times. C, Typical image of representative morphology of the cobblestone areas. D, Frequencies of cobblestone area‐forming cells (CAFC) after the treatment of TNFSF15 at 2 µg/mL for 7 d (n = 12). The experiment was repeated for three times. E, Typical image of TNFSF15‐treated cultures of DAPI^−^CD34^+^CD38^−^CD90^+^CD45RA^−^CD49f^+^ cells in expansion medium for 14 d in single‐cell assay. F, The number of effect wells with expanded cell numbers in the presence or absence of TNFSF15 for 14 d at 2 µg/mL (n = 60). And the experiment was repeated for three times. G, Cell counts of each well of the single‐cell cultures using a BD LSR Fortessa cell analyzer (Becton Dickinson Biosciences); TNFSF15‐treated, number of wells n = 32; vehicle‐treated, n = 13; horizontal bars indicate mean values. (H) Number of CD34^+^ cells in each well of the single‐cell cultures. CD34^+^ cells in the single‐cell cultures were sorted and calculated using a BD LSR Fortessa cell analyzer; TNFSF15‐treated, number of wells n = 25; vehicle‐treated, n = 21; horizontal bars indicate mean values. I, Number of CD34^+^CD49f^+^ cells in each well of the single‐cell cultures

Haematopoietic colony‐forming unit (CFU) assays represent a classical tool for quantifying and evaluating haematopoietic progenitor content which was produced by haematopoietic stem cell within samples. The dysregulation of HSC biology may lead to unbalanced differentiation and could be detected by the presence of single dominant colonies in the CFU assay. Thus, we determined the capacity of TNFSF15 to promote the expansion of haematopoietic progenitor cells by using short‐term haematopoietic colony‐forming unit (CFU) assays.^20^ We cultured CD34^+^ cells from human cord blood for 7 days before plating into semi‐solid culture media for 14 days. All kinds of colonies Burst‐forming unit‐erythroid (BFU‐E), Colony‐forming unit‐erythroid (CFU‐E), Colony‐forming unit‐granulocyte (CFU‐G), Colony‐forming unit‐macrophage (CFU‐M), (Colony‐forming unit‐granulocyte, macrophage (CFU‐GM) and Colony‐forming unit‐granulocyte, erythroid, macrophage, megakaryocyte (CFU‐GEMM) were found in the semi‐solid media based on morphology, and there was no difference in the distribution or morphology of colonies between TNFSF15 treatment and control groups. This indicated TNFSF15 showed no influence on multi‐lineage differentiation potential of HSC. However, the number of colony‐forming unit‐erythroid (CFU‐E), Burst‐forming unit‐erythroid (BFU‐E), Colony‐forming unit‐granulocyte (CFU‐G), Colony‐forming unit‐macrophage (CFU‐M), Colony‐forming unit‐granulocyte, macrophage (CFU‐GM) and Colony‐forming unit‐granulocyte, erythroid, macrophage, megakaryocyte (CFU‐GEMM) were increased to 3.19‐, 4.43‐, 3.32‐, 3.88‐, 2.65‐, 2.14‐ and 2.25‐fold, respectively (Figure 2A and B). Noticeably, the number of colonies formed by CFU‐GEMM cells, which represent more primitive and multi‐potent progenitor cells derived from primary HSC, significantly increased in response to TNFSF15 treatment. This indicated that treatment with TNFSF15 significantly increased the haematopoietic stem and progenitor cells by the colony‐forming capacity assay.

We also assessed the long‐term self‐maintenance capacity of TNFSF15‐treated HSC by using the cobblestone area‐forming cell (CAFC) assay.^21^ We treated CD34^+^ cells for 7 days with TNFSF15 (2 µg/mL), then seeded them on irradiated stromal M_2_‐10B_4_ cells, diluted the cell population weekly and counted cobblestone formations in 5 weeks. The frequency of CAFC was calculated and analysed by Poisson distribution assay. The result indicated that the frequency of HSC occurring in TNFSF15‐treated group (HSC: initiating cells = 1/126) was 3‐fold of that of the control group (HSC: initiating cells = 1/386; *P* < .001; Figure 2C and D).

Given the heterogeneity of haematopoietic cells, isolation of single stem cells is essential for exploring the effects of TNFSF15 on self‐renewal and expansion.^22^, ^23^ DAPI^−^ CD34^+^ CD38^−^ CD45RA^−^ CD90^+^ CD49f^+^ ^19^ HSC cells from human cord blood CD34^+^ cells were singly sorted into 96‐well U‐bottom plates, and then cultured in the presence or absence of TNFSF15 for 14 days in expansion culture medium. The single DAPI^−^ CD34^+^ CD38^−^ CD45RA^−^ CD90^+^ CD49f^+^ HSC cell in each well survived and proliferated was considered as an effect well. After culture, we found that the effect well was significantly increased after being treated with TNFSF15 compared with buffer group in 60 repeat wells (Figure 2F). Moreover, the absolute number of total mononuclear cells, CD34^+^ cells and CD34^+^CD49f^+^ cells in TNFSF15‐treated group increased about 100% compared to vehicle‐treated group (Figure 2G‐I), indicating that the HSC population is efficiently maintained by TNFSF15 during the length of the experiment.

### 3.3 TNFSF15 showed synergistic effect in combination with SR1

**FIGURE 3:**
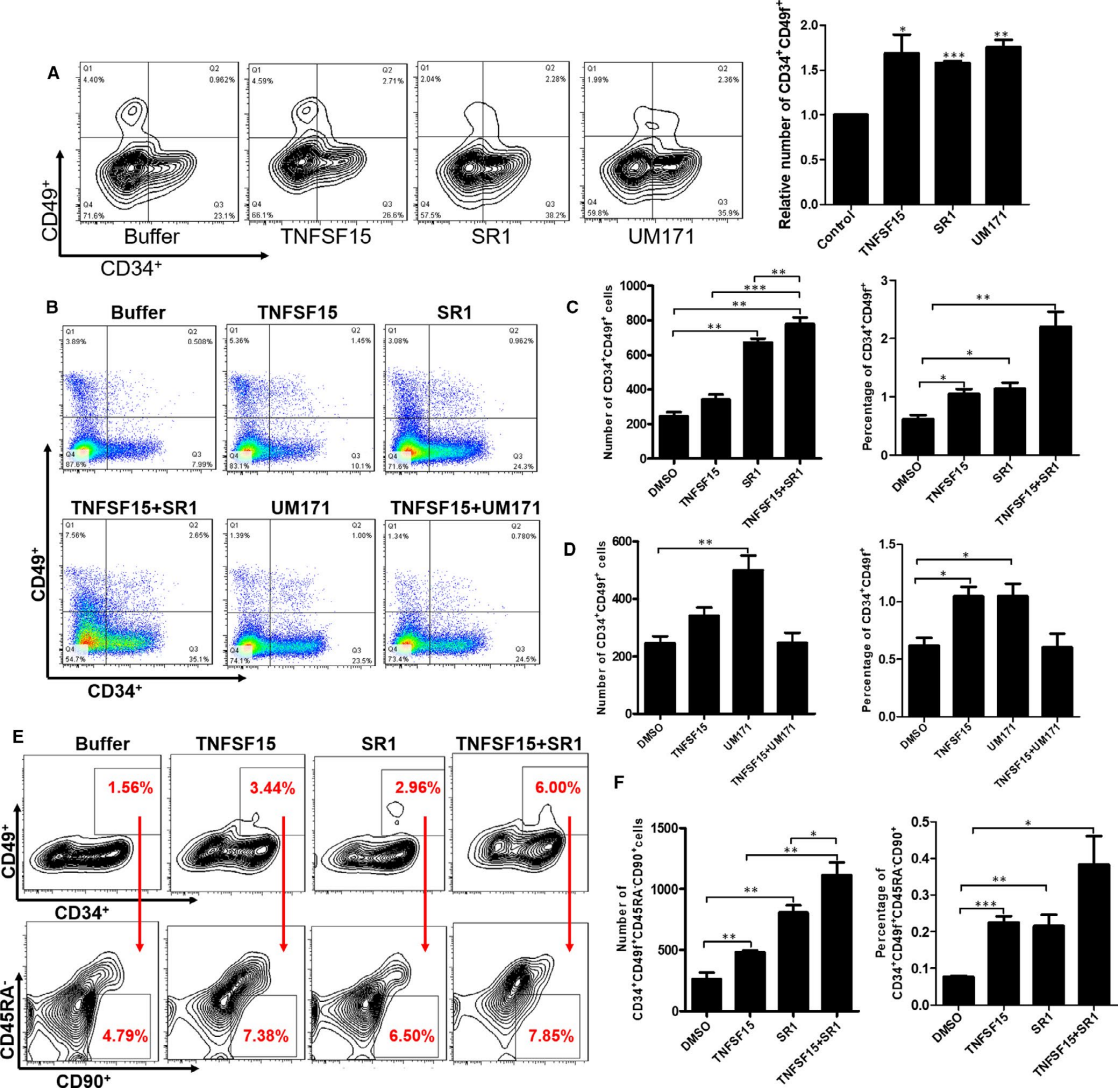
TNFSF15 showed synergistic effect in combination with SR1. A, Representative images of flow cytometry analysis of CD34^+^CD49f^+^ cells after the treatment of TNFSF15, UM171 and SR1 respectively for 7 d in expansion medium. TNFSF15 (2 µg/mL), SR1 (1 µmol/L) and UM171 (35 nmol/L). B, Representative images of flow cytometry after cultured with combination of TNFSF15 and SR1 or UM171. C, The percentage and absolute number of CD34^+^CD49f^+^ cells after cultured with combination of TNFSF15 (2 µg/mL) and SR1 (1 µmol/L); horizontal bar, mean value, n = 4. D, The percentage and absolute number of CD34^+^CD49f^+^ cells after cultured with combination of TNFSF15 (2 µg/mL) and UM171 (35 nmol/L); horizontal bar, mean value, n = 4. E, The representative images of flow cytometry after cultured with combination of TNFSF15 and SR1 for 7 d. F, The percentage and absolute number of CD34^+^CD49f^+^CD45RA^−^CD90^+^ cells after cultured with combination of TNFSF15 (2 µg/mL) and SR1 (1 µmol/L) for 7 d in expansion medium; horizontal bar, mean value, n = 3

Small molecules are emerging as valuable tools for regulating stem cell fates. Recent studies showed that aryl hydrocarbon receptor (AhR) antagonist named SR1^24^ and UM171^25^ with unclear mechanism showed excellent effect on haematopoietic stem cell expansion. TNFSF15 showed almost the same effective on CD34^+^CD49f^+^ cell expansion compared with SR1 and UM171, two commonly used reagents for this purpose (Figure 3A). Moreover, TNFSF15 also exhibited a synergistic effect with SR1 (Figure 3B and C) while showed antagonistic effect with UM171 (Figure 3B and D). To further reveal the combination of TNFSF15 and SR1, we detected the percentage and absolute number of CD34^+^ CD49f^+^CD45RA^−^CD90^+^ after cultured for 7 days which showed a synergistic effect with SR1 (Figure 3E and F).

### 3.4 TNFSF15‐induced enhancement of haematopoietic stem cell engraftment in NOD/SCID or NOG mice

**FIGURE 4:**
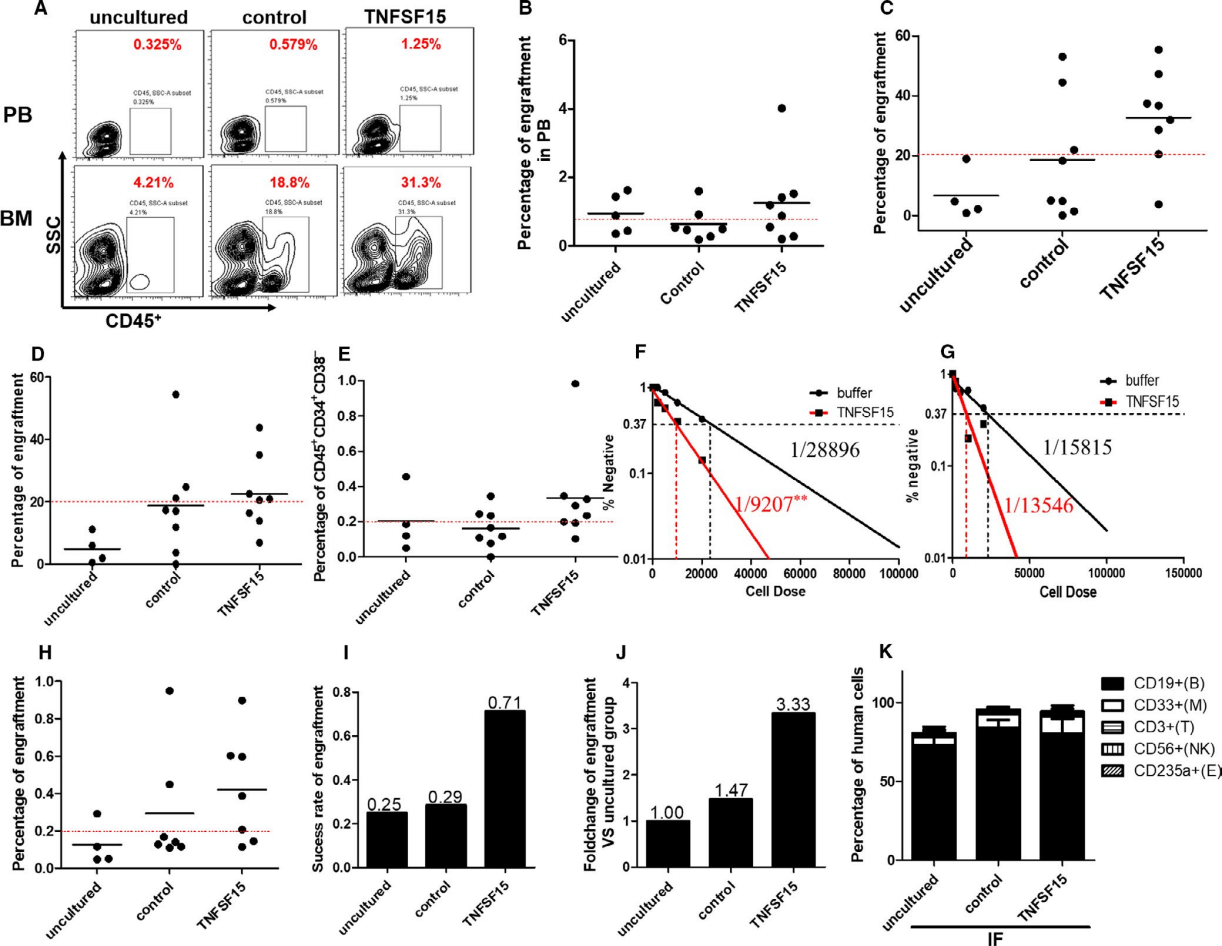
TNFSF15‐induced enhancement of haematopoietic stem cell engraftment in NOD/SCID or NOG mice. A, Representative plots of flow cytometry analysis of human CD45^+^ engraftment in periphery blood and bone marrow of NOG mice engrafted with human UCB‐HSC. B, Percentages of engrafting human cells (CD45^+^) in periphery blood of the experimental animals 12 wk after the implantations; horizontal bars indicate mean values (n = 5 for uncultured group, n = 7 for control group and n = 8 for TNFSF15 group). C, Percentages of engrafting human cells (CD45^+^) in the bone marrows of the experimental animals 12 wk after the implantations; horizontal bars indicate mean values (n = 4 for uncultured group, n = 8 for control group and n = 8 for TNFSF15 group). D, Percentages of engrafting human cells (CD45^+^) in the untreated tibiae of the experimental animals 12 wk after the implantations; horizontal bars indicate mean values (n = 4 for uncultured group, n = 8 for control group and n = 8 for TNFSF15 group). E, Percentages of CD45^+^CD34^+^CD38^−^ cells in the bone marrows of the experimental animals 12 wk after the implantations; horizontal bars indicate mean values (n = 4 for uncultured group, n = 8 for control group and n = 8 for TNFSF15 group). F, Frequencies of SCID mice repopulation cell measured by limiting dilution assay in NOD/SCID mice in the presence or absence of TNFSF15 (2 µg/mL) for 7 d in expansion medium. Four cell concentrations were set in each group (initiating CD34^+^ cells: 2 × 10^4^ cells [n = 7], 1 × 10^4^ cells [n = 7], 5000 cells [n = 7] and 2000 cells [n = 10]). The frequency of SRC cells was calculated by L‐Calc software for limiting dilution assay. G, Frequencies of SCID mice repopulation cell measured by limiting dilution assay in the untreated tibiae of NOD/SCID mice. H, Percentages of engrafting human cells (CD45^+^) in bone marrow 16 wk after the secondary implantation in NOG mice in the presence or absence of TNFSF15 (2 µg/mL) for 4 d (for uncultured, n = 4 mice per group; for control, n = 7 mice per group; for TNFSF15, n = 7 mice per group); horizontal bars indicate mean values. I, Success rates of engraftment in secondary implantations in bone marrow 16 wk after the implantation in NOG mice. J, Fold changes of engraftment of TNFSF15‐treated in comparison with uncultured or vehicle‐treated cells in bone marrow 16 wk after secondary implantations in NOG mice. K, Percentage of cells in multi‐lineage differentiation in multi‐lineage differentiation including myeloid (CD33^+^), lymphocyte cell (CD19^+^), T lymphoid cell (CD3^+^), erythroid cell (CD235a) and NK cell (CD56) after transplantation

To determine the effect of TNFSF15 on the engrafting capability of TNFSF15‐treated UCB‐HSC, we sorted 300 CD34^+^CD38^‐^CD45RA^−^CD90^+^ cells with or without TNFSF15 treatment for 4 days and transplanted them into the tibia of NOD/Shi‐SCID/IL2Rg^null^ (NOG) mice, using 300 initiating cells per injection. Simultaneously, some NOG mice injected with freshly sorted CD34^+^CD38^−^CD90^+^CD45RA^−^ cells (not cultured) were considered as uncultured control. We found that the percentage of CD45^+^ cells in periphery blood was statistically higher in TNFSF15‐treated group compared to uncultured or vehicle‐treated control groups when analysed after 12 weeks post‐transplantation (Figure 4A and B), and that the percentage of CD45^+^ cells in the injected tibiae in TNFSF15 group was approximately 1.75‐fold of that in the vehicle group (Figure 4A and C). The percentage of CD45^+^ cells in the untreated tibiae was also higher, though marginally, in TNFSF15 group than in vehicle‐treated group (Figure 4D). Furthermore, the percentage of CD34^+^CD38^−^ cells in CD45^+^ cells exhibited a 1.8‐fold increase in TNFSF15 group (Figure 4E). These data suggested that TNFSF15‐treated UCB‐HSC maintains HSC’s haematopoietic reconstitution potential and increased the engraftment of UCB‐HSC.

To measure the frequency of HSC present after culture, we performed a limiting dilution analysis with CD34^+^ cells cultured in TNFSF15 or vehicle control into NOD‐SCID mice. Haematopoietic cells with the capable of haematopoietic reconstitution were considered as NOD‐SCID repopulating cells (SRCs) which represented candidate human HSCs. We found that, 12 weeks post‐transplantation, the frequency of SCID mice repopulation cells (SRC) isolated from the tibiae of the TNFSF15 group was 3.14‐fold of that of the control group (Figure 4F). The SRC in the opposite tibiae in TNFSF15 group was 1.17‐fold of that in the control group (Figure 4G).

To determine whether TNFSF15‐expanded cells are capable of long‐term engraftment, we then performed secondary transplant experiments^26^ with human cells isolated from the bone marrow of the NOG mice previously transplanted with TNFSF15‐treated cells and found a rate of successful secondary engraftment of 0.71 compared with 0.25 and 0.29, respectively, for the uncultured and vehicle groups (Figure 4H and I), a completely successful rate being 1.00. Additionally, the percentage of re‐engrafted TNFSF15‐treated cells is 3.33‐fold of that of the uncultured cells (Figure 4J). There is no significant difference in multi‐lineage differentiation including myeloid (CD33^+^), lymphocyte cell (CD19^+^), T lymphoid cell (CD3^+^), erythroid cell (CD235a) and NK cell (CD56) after transplantation without significant difference compared to uncultured group and control group (Figure 4K, Figure S2).

### 3.5 TNFSF15 facilitates human umbilical cord blood haematopoietic stem cell expansion by Notch signal pathway

**FIGURE 5:**
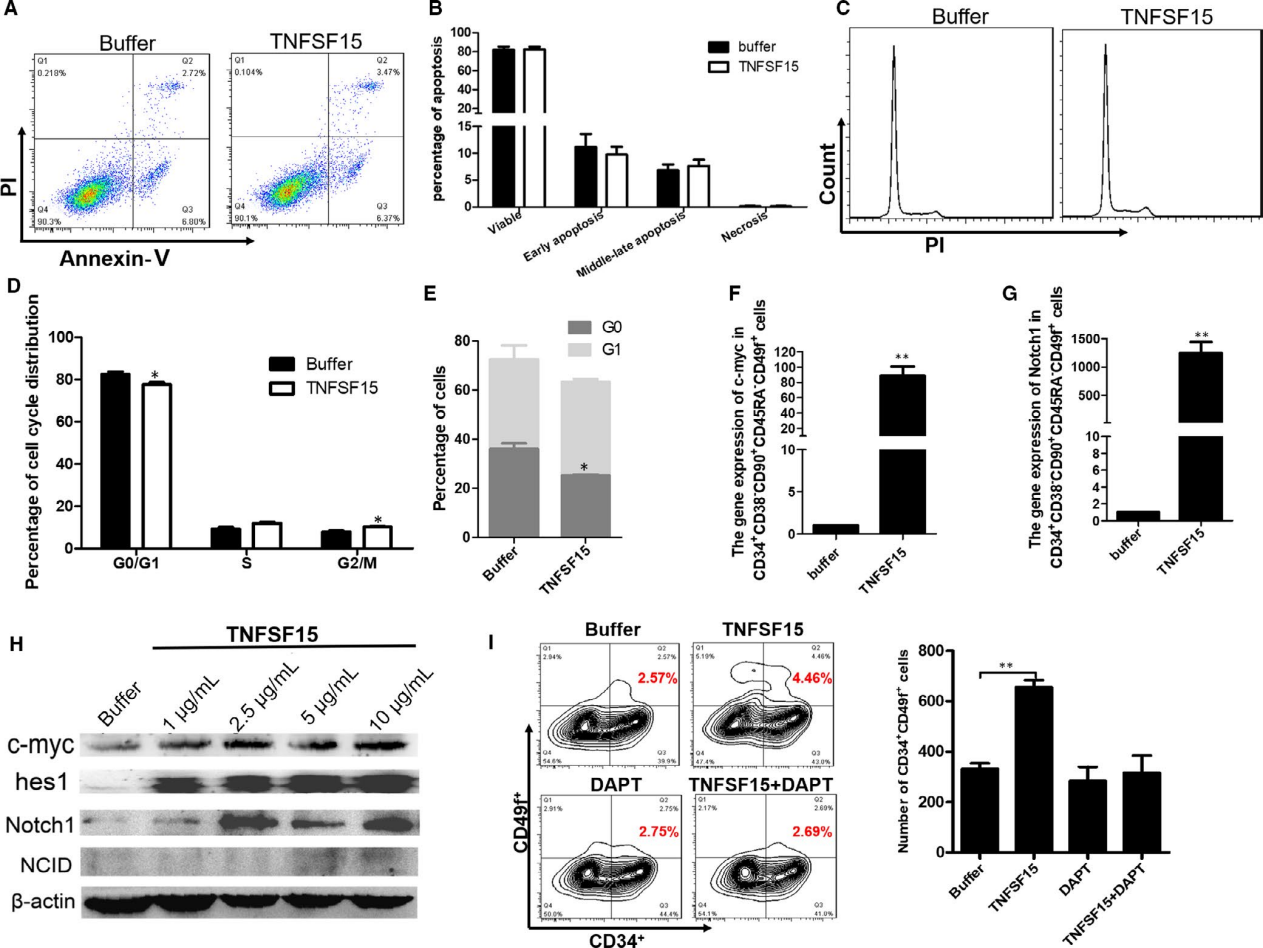
TNFSF15 facilitates human umbilical cord blood haematopoietic stem cell expansion by Notch signal pathway. A, The representative plots of flow cytometry analysis and (B) the statistics of CD34^+^ cell apoptosis rates in the presence or absence of TNFSF15 (2 µg/mL). The experiment was repeated for three times (n = 3). C, The representative plots of flow cytometry analysis and (D) the statistics of CD34^+^ cell cycle distribution of G0/G1, S and G2‐M phase in the presence or absence of TNFSF15 (2 µg/mL) for 7 d in expansion medium (n = 3). The experiment was repeated for three times. E, The percentage of G0 phase was detected in the presence or absence of TNFSF15 (2 µg/mL) for 7 d in expansion medium by Ki67/PI double staining assay (n = 3). F, The gene expression of c‐myc in CD34^+^CD38^−^CD90^+^CD45RA^−^CD49f^+^ cells after the treatment of TNFSF15 (2 µg/mL) for 7 d in expansion medium (n = 3). G, The gene expression of hes1 in CD34^+^CD38^−^CD90^+^CD45RA^−^CD49f^+^ cells after the treatment of TNFSF15 (2 µg/mL) for 7 d in expansion medium (n = 3). H, Western blotting analysis of Notch signal pathway proteins including c‐myc, hes1, Notch1 and NCID in CD34^+^ cells treated with TNFSF15 at indicated concentrations for 7 d in expansion medium. I, The absolute number of CD34^+^CD49f^+^ cells after cultured with combination of TNFSF15 (2 µg/mL) and DAPT (0.5 µmol/L) for 7 d in expansion medium (n = 3)

To analysis the mechanism of TNFSF15 in HSC expansion, cell apoptosis and cell cycle assay were performed. We found TNFSF15 treatment of UCB‐HSC did not impose any significant impact on cell apoptosis rates (Figure 5A and B); rather, TNFSF15‐treated cells exhibited a decrease of the G0/G1 phase and an increase of the G2/M phase when cell cycle distribution was analysed (Figure 5C and D), indicating enhanced cell proliferation. We further performed experiment to distinguish the percentage of the G0 phase from G0/G1 with PI/Ki67 double staining. The results showed the G0 phase after TNFSF15 treatment was decreased clearly (Figure 5E, Figure S1C). To detect the change of genes related in proliferation and differentiation in human HSCs after TNFSF15 treatment, we performed single‐cell PCR assay with 50 CD34^+^CD38^−^CD45RA^−^CD90^+^CD49f^+^ cells sorted from CD34^+^ cells cultured in expansion medium after treatment for 7 days. The expression of 95 genes was detected by PCR assay. The results showed that genes in Notch signal pathway including c‐myc and Notch1 were significantly changed (Figure 5F and G). Since the Notch signalling pathway is reported to be pivotal in the sustenance and proliferation of HSC,^27^, ^28^, ^29^ we determined the relative abundance of each of the Notch signal pathway proteins C‐myc, Hes1 and Notch1 in TNFSF15‐treated UCB‐HSC, and found these signals were significantly up‐regulated compared with vehicle‐treated cells (Figure 5H), indicating Notch signal activation by TNFSF15‐treatment. In order to validate the activating of Notch signal pathway after TNFSF15 treatment, we inhibited Notch pathway with an inhibitor named DAPT. The result of flow cytometry showed that Notch pathway inhibitor inhibited the effect of TNFSF15 (Figure 5I).

## 4 DISCUSSION

Haematopoietic stem cells from human umbilical cord blood (hUCB‐HSCs) have been a significant source of haematopoietic stem cells due to tremendous promise for clinical HSC transplantation.^30^ However, the relatively low number of HSCs in a UCB unit limits its successful widespread transplantation especially in adult recipients. One strategy to overcome this barrier was the application of two partially HLA‐matched UCB units while this aggravated the difficult of HLA‐matched, slow platelet and neutrophil engraftment, and made it much more impossible for auto‐transplantation patients.^31^, ^32^ The other strategy was made effort in searching culture conditions that sustain hUCB‐HSCs expansion ex vivo and developed cellular therapies to improve the outcomes of hUCB‐HSCs transplantation.^30^ Cytokine cocktails including SCF, TPO, FLt3, IL‐3 and IL‐6 were frequently used in hUCB‐HSCs expansion ex vivo which resulted in rapid proliferation of partially differentiated HSC compartment while losing the characteristic of long‐term repopulating ability.^33^ Small molecules such as SR1,^24^ UM171,^25^ CHIR‐911,^34^ valproic acid^35^ and so on were of increasing concern with the effect of promoting HSC self‐renewal, multi‐lineage potency activities and homing, and as to SR1 the engraftment for neutrophils and platelets showed significantly faster than that in patients treated with unmanipulated UCB in clinical trial^36^ which showed attractive prospects, while the effect and the safety needed further identification.^30^ As a result, there were no sufficient means to achieve the expansion of human HSCs in the present culture conditions.

TNFSF15, an endogenous vascular endothelial growth inhibitor (VEGI), is mainly produced by vascular endothelial cells in a normal tissue and modulated vascular homeostasis by inhibiting endothelial cells proliferation. Previously, our group reported that TNFSF15 treatment caused the number of HSC cells in the bone marrow to increase by about three‐fold which prompt us to propose that TNFSF15 may promote HSC production.^16^ To verify our hypothesis, herein we demonstrate that TNFSF15 has the ability to promote UCB‐HSC expansion ex vivo in amplification cultures stimulated by combination of SCF, TPO and Flt3. Our data showed a significant increase of UCB CD34^+^CD49f^+^ cells after cultured for 7 days with the treatment of TNFSF15. Further characterization of the HSCs showed the percentage and absolute number of CD34^+^CD45RA^‐^, CD34^+^CD90^+^, CD34^+^CD49f^+^CD90^+^CD45RA^−^ and CD34^+^CD49f^+^CD90^+^CD45RA^−^CD38^−^ were significantly more preserved after treatment of TNFSF15. The CFUs assay showed that TNFSF15 increased the number of colony‐forming units especially CFU‐GEMM which indicated that TNFSF15 promoted the expansion of multi‐potent progenitors. Although the multiple differentiation potential is responsible to HSC for producing kinds of functional blood cells, the capacity of self‐renewal is critical in maintaining the size of the HSC pool.^3^ The CAFC assay demonstrated that TNFSF15 could promote the self‐renewal of hUCB‐HSCs. In consecutive transplant, experiments with NOG mice and NOD/SCID mice indicated that TNFSF15 promoted the expansion of hUCB‐HSCs that retained multi‐lineage long‐term engraftment. Furthermore, the mechanism study of TNFSF15 for the expansion of hUCB‐HSCs showed that TNFSF15 affected mainly by activating Notch signal pathway.

## 5 CONCLUSION

We demonstrated that TNFSF15 has the ability to promote UCB‐HSC expansion ex vivo through activating Notch signal pathway, and TNFSF15‐treated HSCs are able to sustain bone marrow engraft. Our research suggested that TNFSF15 may thus be useful for acquiring HSC from umbilical cord blood for the treatment of haematological malignancies and degenerative diseases.

## CONFLICT OF INTEREST

The authors confirm that there are no conflicts of interest.

## AUTHOR CONTRIBUTION

**Yahui Ding:** Data curation (lead); Investigation (lead); Methodology (lead); Software (lead); Validation (equal); Visualization (lead); Writing‐original draft (lead); Writing‐review & editing (equal). **Shan Gao:** Data curation (supporting); Investigation (supporting); Methodology (supporting); Supervision (supporting); Visualization (equal). **Jian Shen:** Methodology (supporting); Software (supporting). **Tianran Bai:** Conceptualization (supporting); Methodology (supporting). **Ming Yang:** Methodology (supporting). **Shiqi Xu:** Data curation (supporting). **Yingdai Gao:** Funding acquisition (equal); Methodology (supporting); Project administration (equal); Supervision (equal). **Zhisong Zhang:** Conceptualization (equal); Data curation (supporting); Formal analysis (equal); Funding acquisition (equal); Investigation (equal); Project administration (equal); Supervision (equal). **Luyuan Li:** Conceptualization (equal); Formal analysis (equal); Funding acquisition (equal); Investigation (equal); Methodology (equal); Project administration (lead); Writing‐review & editing (lead).

## ETHICS APPROVAL AND CONSENT TO PARTICIPATE

All the animals were purchased from the Chinese Academy of Sciences (Beijing, China). All animal experimental protocols were approved by the Institutional Animal Care and Use Committee (IACUC), Institute of Hematology and Blood Diseases Hospital, CAMS/PUMC. The UCB collection made according to the Declaration of Helsinki in 1975 by professional gynaecologist. And all experimental procedures have been done according to the institutional guidelines.

## CONSENT FOR PUBLICATION

All authors reviewed and approved the manuscript.

## Supporting information

Fig S1‐S2

## Data Availability

All data generated or analysed during this study are included in this published article. The data sets analysed during the current study available from the corresponding author on reasonable request.
