# Supplementary material for: TNFSF15 facilitates human umbilical cord blood haematopoietic stem cell expansion by activating Notch signal pathway
Source: J Cell Mol Med. 2020 Sep 10;24(19):11146–57. doi: 10.1111/jcmm.15626 (PMC7576288; doi:10.1111/jcmm.15626)
Supplement: Supplementary file 1 — Fig S1‐S2 [file JCMM-24-11146-s001.docx]

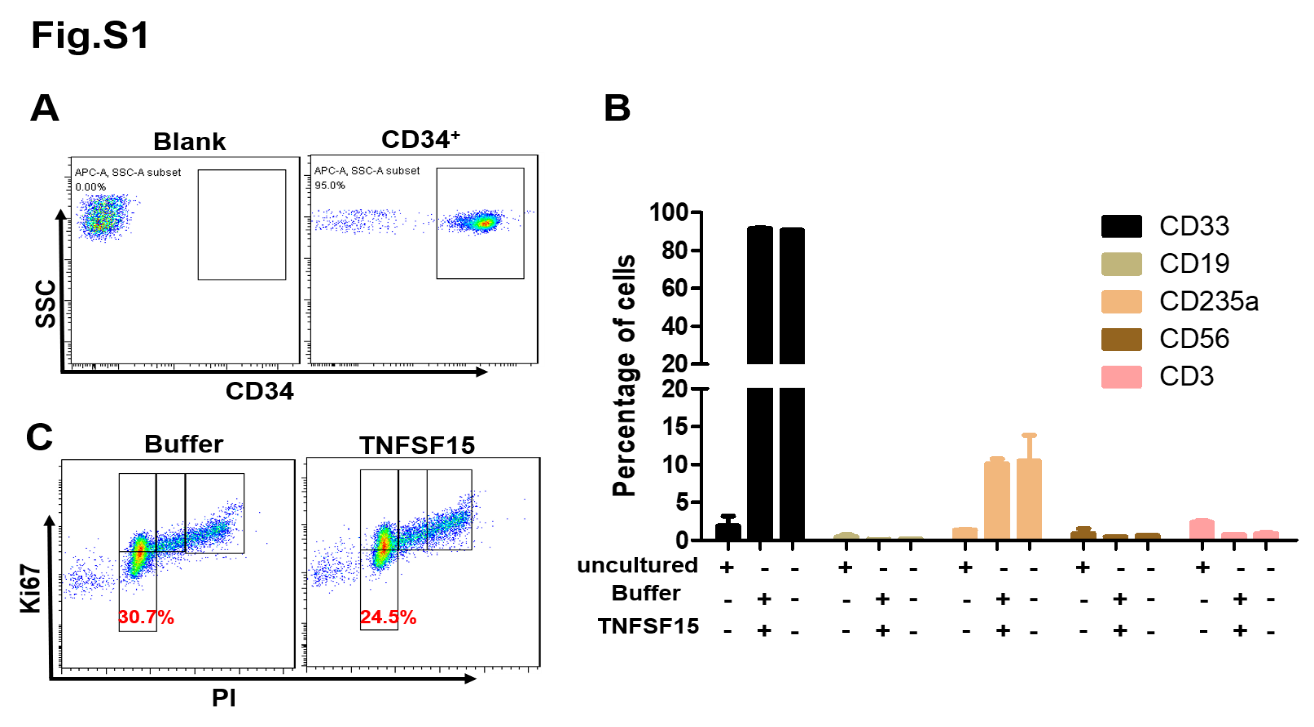


Fig. S1: (A) The purity of CD34^+^ HSC detection after magnetic sorting from Umbilical cord blood. (B) The capacity of differentiation into different lineages including myeloid cell (CD33), erythroid cell (CD235a), lymphocyte cell (CD19), T cell (CD3) and NK cell (CD56) were analyzed in the presence of SCF, TPO, and Flt3L at 100 ng/mL with or without TNFSF15 (2 µg/mL) treatment for 7 days compared with uncultured group. (C) The percentage of the G0 phase in expansion medium with or without TNFSF15 (2 µg/mL) treatment for 7 days with PI/Ki67 double staining.


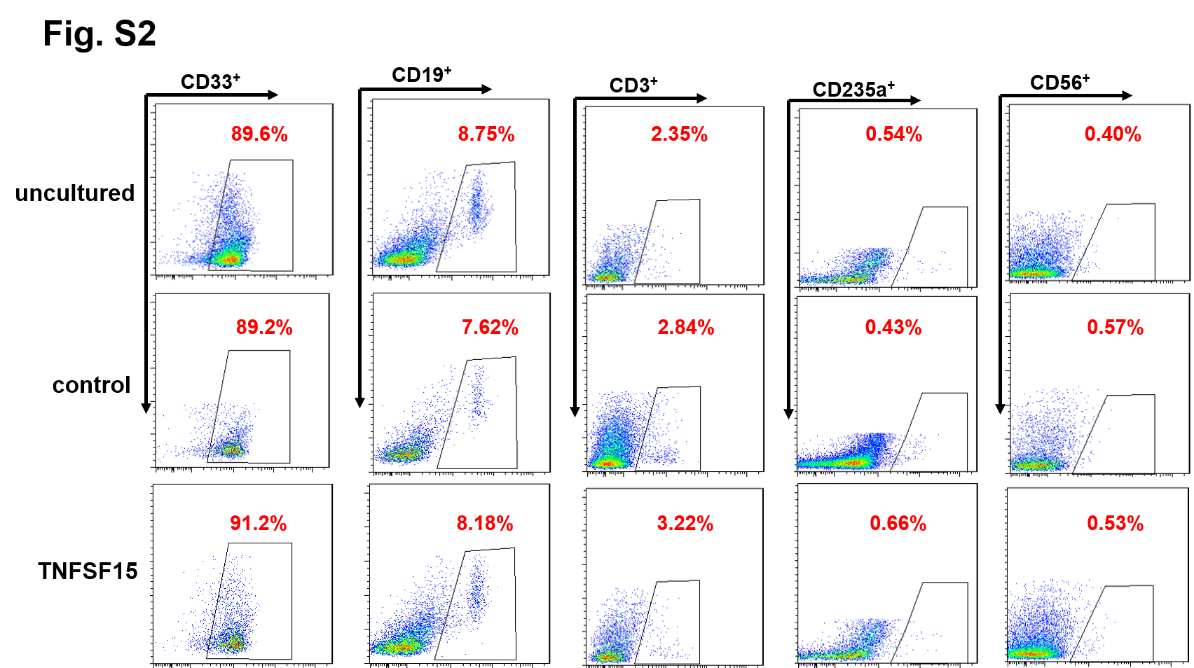


Fig. S2: The flow cytometry images of multi-lineage differentiation into different lineages after transplantation including myeloid cell (CD33), lymphocyte cell (CD19), T cell (CD3), erythroid cell (CD235a), T cell (CD3) and NK cell (CD56).
